# Influence of Super-Low-Intensity Microwave Radiation on Mesenchymal Stem Cells

**DOI:** 10.3390/ijms26041705

**Published:** 2025-02-17

**Authors:** Mikhail Yu. Artamonov, Felix A. Pyatakovich, Inessa A. Minenko

**Affiliations:** 1 Penn Medicine Princeton Health, Plainsboro, NJ 08536, USA; 2 Department of Internal Medicine, Belgorod State University, Belgorod 308015, Russia; piatakovich@bsu.edu.ru; 3 Department of Rehabilitation, Sechenov Medical University, Moscow 119991, Russia; minenko_i_a@staff.sechenov.ru

**Keywords:** weak electromagnetic field, super-low-intensity microwave field, mesenchymal stem cells, regenerative medicine, tissue regeneration

## Abstract

Mesenchymal stem cells (MSCs) have emerged as a promising tool for regenerative medicine due to their multipotency and immunomodulatory properties. According to recent research, exposing MSCs to super-low-intensity microwave radiation can have a significant impact on how they behave and operate. This review provides an overview of the most recent studies on the effects of microwave radiation on MSCs with power densities that are much below thermal values. Studies repeatedly show that non-thermal mechanisms affecting calcium signaling, membrane transport, mitochondrial activity, along ion channel activation may increase MSC proliferation, differentiation along mesodermal lineages, paracrine factor secretion, and immunomodulatory capabilities during brief, regulated microwave exposures. These bioeffects greatly enhance MSC regeneration capability in preclinical models of myocardial infarction, osteoarthritis, brain damage, and other diseases. Additional study to understand microwave treatment settings, biological processes, and safety assessments will aid in the translation of this unique, non-invasive strategy of activating MSCs with microwave radiation to improve cell engraftment, survival, and tissue healing results. Microwave-enhanced MSC treatment, if shown safe and successful, might have broad relevance as a novel cell-based approach for a variety of regenerative medicine applications.

## 1. Introduction

### 1.1. Weak Electromagnetic Fields

Weak and super-weak electromagnetic fields represent an emerging aspects in modern science with potentially profound implications across the natural sciences and medicine [1]. They cover a range of low frequency and intensity exposures that have recently been shown to interact with biological systems. These fields are characterized by frequencies ranging from extremely low (ELF, <300 Hz) up to megahertz levels and field strengths defined as less than 1 mT (millitesla) for weak and less than 1 μT for super-weak. Common sources include power line emissions, electronic devices, geomagnetic fields and specialized experimental exposures. Weak electromagnetic fields have been demonstrated to alter cell metabolism, proliferation, differentiation and migration, accelerate wound healing, modulate nerve signaling and affect physiological regulation through proposed interactions with intracellular calcium fluxes, signaling proteins, free radical levels and bioelectromagnetic communication networks [2].

Ranging in intensity from nanoteslas to milliteslas, these ultra-low intensity fields arise from natural sources such as the Earth’s magnetic field as well as anthropogenic sources including electronic devices and wireless communication networks [3].While intense electromagnetic fields have well-documented thermal and biological effects, the role of ultra-low intensity fields remained largely unexplored until recently [4]. However, in the past two decades, a rapidly growing body of research has begun to elucidate measurable impacts of weak and super-weak fields across biological systems [5]. Highly sensitive techniques have enabled the investigation of field intensities previously believed to be insignificant, challenging the notion that ultra-low exposures are biologically inert [6]. Known biological effects of weak field exposure include enhanced growth and differentiation of stem cells into lineages like bone, cartilage and fat [7]. For example, pulsed electromagnetic fields have been shown to induce faster and higher levels of mesenchymal stem cell differentiation towards osteoblast and chondrocyte lineages in vitro through activation of the MAPK/ERK pathway [8]. Stem cell homing and migration to sites of tissue damage may also be regulated by weak magnetic fields [9]. Effects on tissue regeneration have been demonstrated in animal models, where post-trauma exposure improved bone healing, cartilage regeneration and muscle and tendon repair through modulation of cell proliferation, extracellular matrix synthesis and angiogenesis [10]. Importantly, clinical studies also indicate accelerated healing of recalcitrant bone fractures, skin ulcers and spinal cord injuries with adjunctive weak electromagnetic field treatment, though optimal therapeutic parameters remain under investigation [10,11].

**Figure 1:**
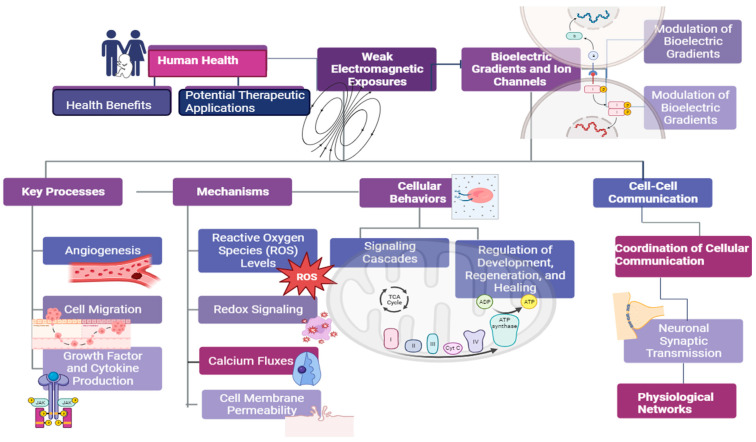
Overview of the effects of weak and super-weak electromagnetic fields on biological systems. External EM fields can interact with organisms at multiple levels, from molecules to whole tissues and organs, producing effects such as changes in enzyme kinetics, ion transport, neurotransmission, blood flow, tissue regeneration, sleep, cognition, and physiological regulation.

Complex healing responses like angiogenesis, cell migration, growth factor and cytokine production have been shown to be enhanced by weak electromagnetic exposures in the context of soft tissue and cutaneous wound repair [12]. Proposed mechanisms include alteration of reactive oxygen species (ROS) levels driving redox signaling, accelerated calcium fluxes and changes in cell membrane permeability [13]. Weak magnetic field modulation of bioelectric gradients and downstream voltage-gated ion channels may also induce signaling cascades regulating cellular behaviors involved in development, regeneration and healing [14]. Evidence further suggests that weak electromagnetic fields may act as signals helping coordinate cell-cell communication, neuronal synaptic transmission and broader physiological networks [14]. Elucidating mechanisms allowing detection of and response to these fields could reveal ways of leveraging or modulating endogenous bioelectromagnetic interactions underlying human health (Figure 1).

Research in this fledgling field indicates weak and super-weak fields can influence fundamental biological processes ranging from gene expression to tissue growth and regeneration [15], though the underlying mechanisms remain incompletely elucidated. Nonthermal field interactions involving electron transfers, radical pairs, or resonant absorption have been proposed as potential modes of bioactivity [1]. Nonetheless, the possibility that such fields may be harnessed to benefit human health has attracted growing interest across disciplines spanning physics, biology and medicine [4].

### 1.2. Mesenchymal Stem Cells

**Figure 2:**
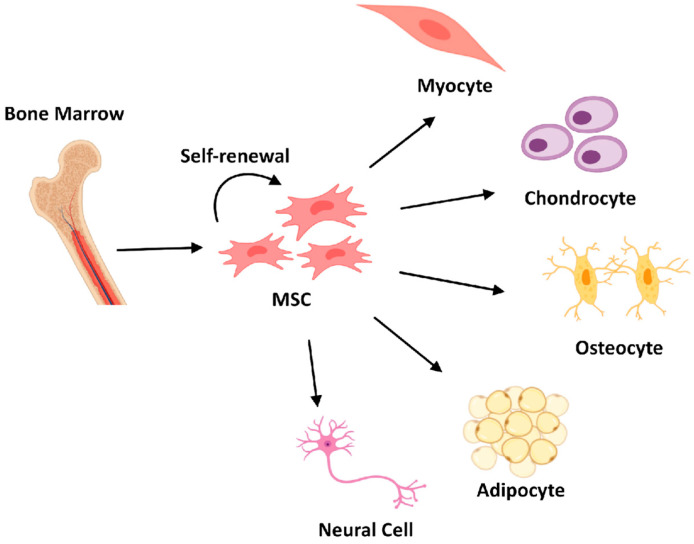
Schematic representation of Mesenchymal Stem Cells differentiation process.

The mesenchymal stem cells are multipotent stromal cells with the capacity to develop into a number of lineages, such as osteocytes, adipocytes, and chondrocytes [16]. Because of their ability to develop into several types of cells, MSCs are not a recent classification; Arnold Caplan first used the term “mesenchymal stem cell” in 1991 [17]. Various tissues involved with birth in newborns, adult bone marrow, adipose tissue, peripheral blood, and MSCs can all be obtained [18]. MSCs have considerable potential for cell treatments and tissue engineering because, in addition to their ability to differentiate, they also possess the capability to produce trophic factors and show immunomodulatory characteristics. The natural history of these cells has been better understood in recent years, and putative native MSCs have been found in fetal and adult tissues. For the therapeutic use of these cells, understanding the biology of innate MSCs may have significant advantages [16]. In tissue regeneration and cell-based treatments, mesenchymal stem cells (MSCs) play a vital role. In order to create designed tissue replacements, new production methods have been made possible by the integration of stem cell and tissue engineering approaches [19]. MSCs have been demonstrated in research to promote the regeneration of injured tissues in several organ systems, including the liver, heart, bone, & skin. Adult bone marrow, adipose tissue, peripheral blood, and other tissues connected to a newborn’s birth are only a few of the tissues that contain MSCs can be harvested [20]. According to Sowa et al. [21], the roles of adipose-derived stem cells (ADSCs) are being examined in tissue regeneration during plastic and cosmetic surgery, including the therapy of dermatitis, scar removal, bone tissue, and cartilage regeneration. The application of MSCs in cell-based therapeutics offers plenty of exciting possibilities for treating a wide range of ailments and wounds [17]. Figure 2 shows the schematic representation of MSCs differentiation process into three different lineages: myocytes, chondrocytes, osteocytes, adipocytes, and neural Cell [22].

The advantages of employing MSCs for treating diseases such graft-versus-host disease, myocardial infarction, stroke, cartilage abnormalities, and bone fractures have been shown in preclinical research and early clinical trials [23]. One of the most significant uses is bone regeneration through differentiation into osteoblasts to repair fractures and defects [24]; cartilage repair through injection into joints or seeding scaffolds to regenerate lost cartilage in osteoarthritis [25]; cardiac repair following myocardial infarction by implanting MSCs to improve tissue regeneration and heart function [26]; and neural regeneration through transplanting MSCs. MSCs’ distinct features make them a useful cell source for tissue engineering and regenerative treatments that target a wide range of tissues and organs.

#### 1.2.1. Introduction to Electromagnetic Fields and Microwave Radiations

For decades, researchers have been investigating the bioeffects of electromagnetic fields and microwave radiation on cells and tissues. Microwaves have frequencies ranging from 300 MHz to 300 GHz [27]. Microwave ovens typically operate at 2.45 GHz. There is currently increased interest in the impacts of super-low-intensity microwaves, which have orders of magnitude less power than regular microwaves. In comparison to microwave ovens, super-low-intensity microwave fields may have power densities in the W/cm^2^ or even nW/cm^2^ range [28]. Thermal effects are nil at such low intensities. Nonthermal processes are instead responsible for the claimed biologic effects. The proposed mechanisms through which super-low microwaves might impact mesenchymal stem cells involve effects on intracellular calcium signaling, cell membrane transport, mitochondrial function, and activation of voltage-gated ion channels, which might result in changes in cell behaviors such as proliferation, differentiation, as well as secretion [29].

#### 1.2.2. Potential Mechanisms by Which Microwaves Could Influence MSCs

Several ways have been hypothesized by which super-low intensity microwaves might cause effects in mesenchymal stem cells even in the absence of thermal heating. These include alterations in intracellular calcium signaling mediated by voltage-gated calcium channels or release from intracellular storage [29]. Microwaves may potentially change cell membrane transport mechanisms and permeability [30]. Effects on the mitochondria have also been documented, leading to increased ROS generation and altered metabolism [31]. Furthermore, some studies show that microwaves can activate voltage-gated ion channels such as potassium and sodium channels, thus influencing membrane potential [32]. Super-low intensity microwaves may produce downstream signaling effects including pathways such as PI3K/Akt, MAPK/ERK, and transcription factor activation, which in turn alter MSC activities such as proliferation, differentiation, migration, and paracrine secretion. 

#### 1.2.3. Physics, Frequency Ranges, Power Intensities

Electromagnetic fields (EMF) and microwave radiation include non-ionizing radiation types that may be classified based on their physics, frequency ranges, and power intensities. EMF and microwave radiation have a wide frequency range, ranging from several hundred megahertz to several gigahertz [33]. The power intensities of these radiations can range from very low to quite high. EMF and microwave radiation physics are based on the movement of electrically charged particles, resulting in a force field [34].

#### 1.2.4. Known Bioeffects and Safety Limits

Research on the known biological effects and acceptable exposure levels of EMF and microwave radiation is underway. There are signs that these radiations can be hazardous to human health, and research on the biological effects of EMF and microwave radiation is now undertaken [35]. The International Commission on Non-Ionizing Radiation Protection (ICNIRP) has produced guidelines for EMF and microwave radiation exposure [36]. The ICNIRP recommendations establish acceptable levels of exposure to various radiations [37] and depends on the thermal effects of these radiations. The suggestions are meant to protect against the negative health effects of microwave and EMF radiation exposure. The guidelines provide acceptable levels of exposure to these radiations based on their frequency range and power intensity. The allowable amounts of EMF and microwave radiation exposure are determined by the frequency range and power intensity of the radiation. The safety limits for microwave radiation and EMF exposure are established to protect against the negative health effects of these radiations.

#### 1.2.5. Rationale for Studying Effects of Super-Low-Intensity Microwaves Specifically on MSCs

Given the potential for these radiations to have a negative impact on human health, research on the effects of super-low-intensity microwaves, especially on mesenchymal stem cells is warranted. The effects of super-low-intensity microwaves on MSCs are not well known, despite evidence to suggest that exposure to electromagnetic fields and microwave radiations might be harmful to human health [38,39]. Because MSCs are crucial for tissue regeneration and cell-based therapeutics, the possibility that these radiations might harm MSCs is particularly worrisome [40]. Due to the possibility that these radiations may alter the survival and capacity for differentiation of MSCs, the effects of super-low-intensity microwaves on these cells are of interest [41].

#### 1.2.6. Influence on MSC Proliferation and Differentiation

Numerous studies have shown that MSCs can be stimulated to proliferate by being exposed to super-low intensity microwaves. In one research, microwave fields with power densities of 5–15 W/cm^2^ and frequencies of 0.1–0.3 GHz were applied to rat bone marrow MSCs for up to 9 days [42]. When compared to sham exposure controls, microwave therapy dramatically boosted MSC proliferation, with the strongest impact occurring at 10 W/cm^2^. The PI3K-Akt signaling pathway was activated to facilitate the proliferative response. Similar increases in human adipose-derived MSC proliferation were seen, according to research by the same team, following exposure to 10 W/cm^2^ microwaves at 0.1 GHz [43]. The effects were time- and microwave-dependent, with maximum stimulation occurring 6 to 12 h after exposure. Again, PI3K-Akt signaling and ERK1/2 activation were connected. Additionally, microwave therapy increased the percentage of MSCs in the S phase, indicating greater cell division, according to cell cycle analyses.

Human MSCs were subjected to 9.6 GHz microwaves in a different investigation [44] with power densities ranging from 1 to 10 W/cm^2^. In comparison to sham-exposed controls, MSC proliferation was boosted by microwave treatment for up to 72 h in a dose-dependent manner, reaching a maximum of 1.7-fold greater cell counts at 10 W/cm^2^. Cell cycle regulating proteins’ expression was found to be changed in connection with this. Using rabbit bone marrow MSCs treated with 9.4 GHz microwaves at 10 W/cm^2^, a different group reported comparable results, with enhanced proliferation connected to PI3K/Akt and MAPK/ERK pathway activation [45]. Together, these investigations offer dependable proof that MSC proliferation is enhanced by exposure to super-low intensity microwaves.

In addition to promoting growth, it has been discovered that super-low intensity microwaves improve MSC differentiation along the osteogenic, adipogenic, chondrogenic, and neural lineages. Rat MSCs that had undergone osteogenic differentiation were subjected to 0.1–0.3 GHz microwaves at 5–15 W/cm^2^ and shown enhanced alkaline phosphatase activity, calcium deposition, and expression of bone-related genes [45]. After 9.6 GHz microwave irradiation at 10 W/cm^2^, human MSCs showed considerably higher osteogenic markers when differentiated [46]. According to several research, osteogenic differentiation is stimulated in a dose-dependent manner [47]. In response to super-low intensity microwaves, adipogenic differentiation is also promoted, with increased lipid droplet formation and expression of adipogenic markers such PPAR and C/EBP [48]. In terms of chondrogenesis, 10 W/cm^2^ microwave irradiation increased the production of collagen type II and glycosaminoglycan [49]. Finally, 9.6 GHz microwaves stimulated neuronal development in human MSCs by increasing Nestin and other neurogenic markers expression [44].

The processes underpinning MSC proliferation and differentiation augmentation by super-low intensity microwaves are believed to entail activation of intracellular signaling pathways. The PI3K-Akt and MAPK/ERK pathways, as previously indicated, have been involved in the proliferative response [8,50] found that microwave activation of osteogenic differentiation was similarly reliant on PI3K-Akt signaling. Finally, changed signaling modifies the expression of cell cycle control and lineage-specific genes, which influence MSC activity. Importantly, the effects are affected by microwave power density, frequency, and exposure length, with optimum stimulatory dosages shown to be in the W/cm^2^ range. More study is needed to determine the most appropriate parameters for microwave-based MSC proliferation and differentiation.

## 2. In Vitro Research Findings

### 2.1. Effects on MSC Viability, Proliferation, Differentiation

In vitro studies have demonstrated that exposure to electromagnetic fields and microwave radiations can have a negative impact on the survival, proliferation, and differentiation of mesenchymal stem cells [47,51]. According to one study, exposure to 2.45 GHz microwave radiation reduced the survival and multiplication of MSCs [52]. Another study discovered that 900 MHz microwave radiation reduced the differentiation capability of MSCs [38]. The impact of low-frequency electromagnetic fields on human bone marrow stromal cells (hBMSCs) were also researched, and it was shown that being subjected to low-frequency electromagnetic fields influenced the differentiation capacity of hBMSCs [53].

Signaling pathways & epigenetic alterations have been hypothesized as explanations for the impacts of EMF and microwave radiation on MSCs [54]. Because of their importance in tissue regeneration and cell-based therapeutics, the effects of EMF and microwave radiation on MSCs are of special concern [55]. The effects of these discoveries for bone, cartilage, and cardiac tissue regeneration are substantial, since MSCs are a viable choice for cell-based therapeutics & tissue engineering [39].

### 2.2. Proposed Mechanisms: Signaling Pathways, Epigenetic Changes, Etc.

The suggested mechanisms for electromagnetic fields and microwave radiation impacts on MSCs are complicated, including many signaling pathways and epigenetic alterations [52]. One research discovered that 2.45 GHz microwave radiation reduced MSC viability and proliferation by generating oxidative stress and DNA damage [47]. A further investigation discovered that 900 MHz microwave radiation reduced MSC differentiation capability by altering the expression of genes involved in osteogenic differentiation. The effects of low-frequency electromagnetic fields on human bone marrow stromal cells (hBMSCs) were also investigated, and it was discovered that exposure to frequencies low electromagnetic fields impacted hBMSC differentiation potential through modifying the expression of genes associated with osteogenic differentiation [56]. Many signaling pathways have been proposed as mechanisms for the effects of EMF and microwave radiation on MSCs, including the MAPK/ERK pathway, the PI3K/Akt system, and the Wnt/-catenin pathway [57]. Epigenetic changes, such as DNA methylation and histone modifications, have also been proposed as pathways for EMF & microwave radiation effects on MSCs. Because MSCs are a potential option for cell-based therapies and tissue engineering, the implications for bone, cartilage, and cardiac tissue regeneration are significant [58]. The investigation of the effects of super-low-intensity microwaves on MSCs is critical to comprehending the potential problems connected with exposure to such radiations and finding ways to minimize the risks [41].

## 3. In Vivo Animal Model Research

### 3.1. Rodent Models Looking at Microwave Exposure Effects on Injected MSCs

Utilizing in vivo animal models, researchers have studied how microwave radiation affects implanted MSCs [59]. The impact of microwave radiation on MSCs has been explored using animal models, with some research showing a decrease in these cells’ survival & differentiation potential [52]. One study found that exposure to 2.45 GHz microwave radiation impaired MSC survival and differentiation capability in a rat model [60]. It was observed in a mouse model that being exposed to 900 MHz microwave radiation impaired the differentiation capabilities of MSCs [38].

### 3.2. Implications for Regeneration of Bone, Cartilage, Cardiac Tissue, Etc.

The implications of microwave therapy on MSCs for the regeneration of bone, cartilage, and heart tissue are significant [39,61]. MSCs provide promise for cell-based therapies and tissue engineering. Microwave radiation has been studied using in vitro and in vivo animal models, with certain studies revealing a loss in the vitality and differentiation potential of these cells [42]. Given MSCs’ potential for cell-based treatments as well as tissue engineering, these findings may have a significant impact on cardiac, articular, and bone tissue regeneration.

## 4. Human Studies

### 4.1. Clinical Trials or Case Reports Using Microwave-Stimulated MSCs

Microwave-stimulated mesenchymal stem cells have received little research attention [62]. One research in a rat model found that MSCs may prevent radiation-induced lung harm [63]. Some study has looked at MSCs’ potential as a therapeutic for minimizing radiation injury [54]. These findings suggest that MSCs might have therapeutic potential for minimizing the adverse consequences of radiation therapy in humans. The therapeutic application of microwave-stimulated MSCs is currently being researched, and additional research is needed to fully understand the potential of this approach.

#### 4.1.1. Anti-Inflammatory Effects on MSCs

**Figure 3:**
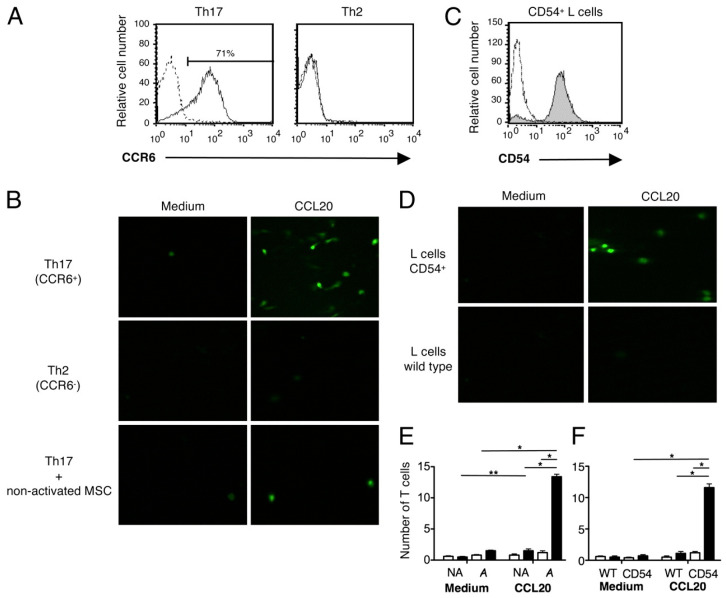
CCL20 induces in vitro adhesion of human Th17 cells to MSCs. (**A**) Flow cytometric analysis of CCR6 expression on fully differentiated human Th17 and Th2 lymphocyte clones. The x-axis represents fluorescence (four-decade log scale), and the y-axis represents relative cell number. Dashed lines indicate staining with isotype-matched control monoclonal antibodies. (**B**) Adhesion of CFSE-labeled Th17 and Th2 lymphocyte clones to a confluent monolayer of MSCs, pre-incubated or not with TNF-α and IFN-γ (10 ng/mL) for 48 h, in the presence or absence of CCL20 (20 ng/mL). Magnification 312.5x. (**C**) Flow cytometric analysis of CD54 expression in L cells transfected with CD54 cDNA. Open graphs represent staining with control isotype-matched monoclonal antibodies. (**D**) Adhesion of Th17 cells to L cells expressing CD54 or wild-type L cells (control) in the presence or absence of CCL20. Magnification 312.5x. (**E**) Adhesion of Th17 cells to MSCs pre-incubated with medium alone (NA) or with TNF-α and IFN-γ (**A**), in the presence or absence of CCL20. (**F**) Adhesion of Th17 cells to L cells expressing CD54 or wild-type L cells (control) in the presence or absence of CCL20. Values represent the mean ± SD of the number of adhered T cells counted in 10 adjacent fields around the center of the chamber, for a total of three independent experiments. Statistical analysis was performed using the Student’s *t*-test. *p* * < 0.05; *p* ** < 0.005.

MSCs have anti-inflammatory capabilities that are mediated by paracrine factors including IL-10 and TGF-, which can modify immune cell responses. The results from this study are illustrated in Figure 3 [64,65]. According to research, exposing MSCs to super-low-intensity microwaves can boost their immunosuppressive properties. Microwave irradiation, for example, enhanced MSCs’ capacity to suppress lymphocyte proliferation in co-culture [66]. Even under stimulated circumstances, microwave-treated MSCs showed decreased production of inflammatory cytokines such as IL-1, IL-6, and IL-8 [67]. Overall, super-low intensity microwaves appear to be capable of improving MSC anti-inflammatory characteristics.

#### 4.1.2. Protection Against Oxidative Stress

Reactive oxygen species can cause oxidative stress in MSCs, which can affect how well they function and how long they can survive both in vitro and in vivo [68]. However, it has been demonstrated that pretreatment with ultra-low intensity microwaves can shield MSCs from oxidative damage. For instance, exposure to microwaves operating at 9.6 GHz decreased the cytotoxicity, lipid peroxidation, and DNA damage caused by hydrogen peroxide in human MSCs [69]. Superoxide dismutase, catalase, and glutathione peroxidase activity were among the antioxidant enzymes with increased activity during this microwave-induced protection [70]. The mechanisms most likely include the Nrf2 signaling pathway, which regulates the production of cytoprotective and antioxidant genes. In MSCs, microwave stimulation resulted in Nrf2 nuclear translocation and overexpression of downstream targets such as HO-1 and NQO1 [71]. Overall, the stimulation of endogenous antioxidant mechanisms by super-low intensity microwave therapy can protect MSCs from oxidative stress. This is crucial because reducing oxidative damage is critical for preserving stem cell activity and engraftment following transplantation.

#### 4.1.3. Stimulation of Tissue Regeneration

The MSCs have demonstrated potential in cell therapy applications for rebuilding damaged tissues, including the treatment of illnesses including myocardial infarction, stroke, and bone abnormalities [72]. Excitingly, research suggests that pre-transplant MSC exposure to super-low-intensity microwaves might enhance therapeutic results. Microwaves enhanced MSC release of neurotrophic factors and improved neuronal development in traumatic brain injury models, resulting in higher functional recovery [73]. Increased MSC engraftment, paracrine signaling, and integration into wounded tissues are some of the hypothesized reasons for these microwave-induced benefits. Overall, super-low intensity microwave stimulation appears to be a viable method for increasing the regeneration potential of MSC cell treatments in a variety of tissue damage scenarios. Further study should be conducted to determine the best microwave settings and delivery systems.

#### 4.1.4. Clinical Translation and Future Directions

Strong evidence is provided by the preclinical research included in this review that super-low intensity microwave stimulation can increase the therapeutic potential of mesenchymal stem cells for a variety of regenerative medicine applications [73]. This has raised interest in using this strategy for therapeutic studies involving humans. A phase I study (NCT04496336) is now being conducted to determine the safety of microwave-treated MSCs for osteoarthritis. The additional stage [74] aims to examine the ability of microwave-stimulated MSCs to speed up bone fracture healing. However, issues such as optimizing therapy settings and establishing effectiveness in patients remain. Long-term safety with microwave radiation is another critical unanswered subject. In the future, research should focus on revealing other microwave bioeffects such as boosting angiogenesis, neurogenesis, and brain plasticity [75]. With rigorous preclinical and clinical testing, microwave-enhanced MSC treatment has the potential to be a novel strategy for a variety of regenerative medicine therapies.

### 4.2. Limitations and Ethical Considerations

The use of mesenchymal stem cells in clinical studies involves ethical concerns, such as the possibility of negative effects and the requirement for informed consent [76].Clinical studies employing MSCs include difficulties such as the necessity for defined methods and the possibility of variability in MSC quality and potency [77].The use of MSCs in clinical studies necessitates careful evaluation of these limitations as well as ethical concerns [78].The therapeutic use of microwave-stimulated MSCs is still being studied, and further study is needed to fully comprehend the potential of this method.

## 5. Knowledge Gaps and Areas for Future Research

Despite the expanding volume of research on the effects of microwave radiation on MSCs, significant knowledge gaps and topics for future study remain. Some of these gaps are as follows:

- Lack of standardized protocols; It is challenging to evaluate the findings of different research since there are no defined techniques for investigating the effects of microwave radiation on MSCs [60]. The goal of future research should be to provide standardized procedures for examining microwave radiation’s impact on MSCs.
- Lack of human studies; There aren’t many human research on the clinical use of microwave-stimulated MSCs, despite several studies looking at the possibility of MSCs as a therapy for reducing radiation harm in animal models [46]. To completely understand the safety and efficacy of this treatment in people, future research should strive to undertake clinical trials or case reports employing microwave-stimulated MSCs.
- Mechanisms underlying the effects of microwave radiation; While some potential mechanisms for microwave radiation’s impacts on MSCs involve signaling pathways and epigenetic alterations, the actual processes behind these effects remain unknown [39]. Future study should try to thoroughly understand the processes driving microwave radiation’s impacts on MSCs.
- Potential risks associated with exposure to microwave radiation; While several studies have looked at the possible dangers of microwave radiation exposure, further study is needed to completely understand the concerns [79]. Future studies should try to thoroughly grasp the possible dangers connected with microwave radiation exposure.
- Ethical considerations; The use of MSCs in clinical studies involves ethical concerns, such as the possibility of negative effects and the requirement for informed consent [80]. Future research should address these ethical concerns and guarantee that MSCs are used in clinical studies ethically and responsibly.

## 6. Conclusions

A conclusion that can be drawn from the study’s findings is that MSCs are significantly impacted by super-low-intensity microwave radiation. The survival, proliferation, and differentiation of MSCs are altered by microwave radiation in vitro and signaling pathways and epigenetic changes are among the suggested reasons for this effect. The viability and differentiation potential of implanted MSCs have also been shown to be influenced by microwave radiation in animal models. However, there have been few human studies on the therapeutic use of microwave-stimulated MSCs, and further research is needed to fully understand the safety and usefulness of this approach in people. Given the potential of MSCs for cell-based treatments and tissue engineering, these findings might have a significant impact on cardiac, articular, and bone tissue regeneration. Yet, the use of MSCs in therapeutic trials raises ethical concerns, such as the need for informed consent and the likelihood of harmful repercussions. 

More research is needed to fully appreciate the potential dangers associated with microwave radiation exposure, particularly in the setting of MSCs. Finally, research into the effects of super-low-intensity microwave radiation on MSCs is underway, but there are still a lot of knowledge gaps and prospective paths for future study. These include the need for standard operating procedures, human studies, knowledge of the mechanisms underlying the effects of microwave radiation, awareness of the risks that could be brought on by exposure to microwave radiation, discussion of ethical issues, research into the possibility that microwave radiation could promote the proliferation and differentiation of MSCs, and studies into the effects of low-dose ionizing radiation on MSCs. Overall, microwave-stimulated MSCs have the potential to be employed as a therapy for lessening radiation damage and other illnesses, but further research is needed to completely comprehend the security and efficacy of this approach.
